# How Accurate Are the ISAAC Questions for Diagnosis of Allergic Rhinitis in Korean Children?

**DOI:** 10.3390/ijerph15071527

**Published:** 2018-07-19

**Authors:** Dong Hyun Kim, Dae Hyun Lim, Mona Samra, Eun Hye Kim, Jeong Hee Kim

**Affiliations:** 1 Department of Pediatrics, Inha University School of Medicine, Incheon 22332, Korea; id@inha.ac.kr (D.H.K.); dhyunlim@inha.ac.kr (D.H.L.); arafaraf8@gmail.com (M.S.); 2 Environmental Health Center for Allergic Rhinitis, Inha University Hospital, Ministry of Environment, Incheon 22332, Korea; ehkim@allergycenter.go.kr

**Keywords:** allergic rhinitis, prevalence, validity, comorbidity

## Abstract

*Background:* The aims of this study were to investigate the prevalence of allergic rhinitis (AR) and the accuracy of the International Study of Asthma and Allergies in Childhood (ISAAC) questions for diagnosis of AR, in Korean children. *Methods:* Students that participated in an allergic disease prevalence survey in 2010–2017 were evaluated (*n* = 18,425) using questionnaires and a skin prick test (SPT). Age−stratified (5−7, 8−10, 11−13, 14−16 years) prevalence of four rhinitis questions, accuracy of the questions for AR, and proportion of comorbidities in the AR and non-AR (NAR) groups were evaluated. *Results:* The proportion of students responding to the questionnaire that ever had symptoms of AR since birth, that is, the prevalence of “symptom, ever” was 47.6%. Based on the questionnaire and SPT, overall prevalence of AR and NAR were 21% and 26.5%, respectively. The sensitivity, specificity, and accuracy of “symptom, ever” were 57.5%, 58.4%, and 58.1%, respectively, and those of “diagnosis, ever”, who had ever been diagnosed with AR, were 39.8%, 76.9%, and 63.4%, respectively. Questionnaire−based asthma, atopic dermatitis, and food allergy were significantly associated with the AR group compared to the NAR group. *Conclusions:* Since the AR accuracy of the questionnaire is about 60%, it should be considered that the questionnaire based survey overestimates the true prevalence of AR in Korean children.

## 1. Introduction

The term rhinitis indicates nasal dysfunction that causes nasal itching, sneezing, rhinorrhea, or nasal blockage. Allergic rhinitis (AR) is defined as IgE-mediated nasal inflammation confirmed by sensitization to inhaled allergens, and non−allergic rhinitis (NAR) is defined by rhinitis but an allergic and an infectious etiology are excluded [1].

The prevalence of AR differs from region to region, due to differences in living environments, and the different allergens involved. The International Study of Asthma and Allergies of Childhood (ISAAC) questionnaires are a widely accepted standardized tool for comparing interregional differences in asthma and allergic disease prevalence [2]. The prevalence of AR has increased in Korea over the past 20 years [3], from 35% to 48% [4,5]. AR and NAR are often difficult to differentiate based on symptoms in children. However, a few epidemiologic studies performed with a combination of a questionnaire for symptoms of rhinitis and an objective measure for IgE in the definition of AR [4,5] suggested that the ISAAC questionnaire alone may overestimate the true prevalence of AR. The prevalence of NAR among children is expected to vary by age, country, regions, and other factors. There are very few studies on the prevalence of NAR.

Thus, the aims of this study were to investigate the prevalence of AR and NAR and determine the accuracy of the ISAAC questions on AR in a general population of Korean children and adolescents based on the symptoms and skin prick test (SPT) results.

## 2. Materials and Methods

### 2.1. Study Population

The subjects included were an unselected general population of 18,425 children and adolescents living in various areas, namely metropolitan (Incheon, Gyeonggi), middle inland (Chungbuk), islands (Gyeonggi Bay archipelago), southern inland (Gwangju), and seaside (Busan) in the Republic of Korea from 2010 to 2017.

### 2.2. Case Definitions and Questionnaire

The AR was defined as having symptoms of rhinitis with one or more positive results of SPT, whereas NAR was defined as rhinitis symptoms with negative SPT results. From the questionnaire, basic personal demographic information was obtained. Rhinitis symptoms were identified through the Korean version of the ISAAC core questions on rhinitis: “Has your child ever had a problem with sneezing or a runny or a blocked nose when he/she did not have a cold or the flu?” (“symptom, ever”), “In the past 12 months, has your child had a problem with sneezing or a runny or a blocked nose when he/she did not have a cold or the flu?” (“symptom, current”), “Has your child ever been diagnosed as AR?” (“diagnosis, ever”), and “In the past 12 months, has your child underwent or been treated for AR?” (“treatment, ever”) [6]. Written informed consent was obtained from all parents and subjects who were older than 6 years.

### 2.3. Allergens

All participants were tested with 18 inhalant allergens. The allergens included house-dust mites (*Dermatophagoides pteronyssinus*, *Dermatophagoides farinae*), pollen (tree: *Cryptomeria* [Japanese cedar], *Betula* [birch], *Quercus* [oak], *Alnus* [alder], *Fagus* [beech], and *Populus* [poplar]; weed: *Ambrosia* [ragweed], *Artemisia* [mugwort], *Humulus* [Japanese hop], and *Chenopodiaceae* [fat hen]; grass: *Cynodon* [Bermuda grass], *Phleum* [timothy grass]), mold (*Aspergillus fumigatus*, *Alternaria tenuis*), and pet (dog, cat). The allergens were manufactured by Allergopharma (Allergopharma GmbH & Co. KG, Hamburg, Germany).

### 2.4. SPT

The SPT was performed by the method described by Kim et al. [5]. Positivity was defined as follows: a wheal with a diameter of > 3 mm, or a wheal ratio of allergen to histamine ≥ 1. The SPTs were carried out on both arms. To exclude errors related to diurnal variation, all the examinations were performed in the morning by trained inspectors.

### 2.5. Diagnostic Accuracy

Using the SPT results as a gold standard, the diagnostic accuracy of the ISAAC questionnaire was calculated utilizing the sensitivity, specificity, positive predictive value, negative predictive value, index for rating diagnostic test, and accuracy as probability of accurate diagnosis. Sensitivity was defined as the proportion of children who positively answered the questionnaire among all those with a positive SPT. Specificity was defined as the proportion of children without symptoms among those with negative SPT results. Positive predictive value (PPV) was defined as the proportion of children with a positive SPT among those acknowledging that they have symptoms in the questionnaire. Negative predictive value (NPV) was defined as the proportion of children whose questionnaire results were concordant with a negative SPT among those with negative questionnaire responses. The accuracy was calculated as follows:

$$\mathrm{Accuracy}\;(\%)\;=\;\frac{TP+TN}{TP+FP+FN+TN}\times 100\;$$

where *T* is true, *F* is false, *P* is positive, and *N* is negative.

### 2.6. Statistical Analysis

Symptom prevalence rates were analyzed for all participants and stratified by age−group and sex. Differences between categorical variables were assessed by the Pearson χ^2^ test. Association between other allergic diseases and comparisons between the AR and NAR groups were further evaluated by logistic regression models with an adjusted odds ratio (aOR) and a 95% confidence interval (95% CI). All of the statistical data were calculated by using SAS version 9.4 (SAS Institute Inc., Cary, NC, USA).

### 2.7. Ethics Statement

The institutional review board of Inha University Hospital approved this study (IRB no.; 11-12, 12-05, 2015-09-007, 2017-02-019).

## 3. Results

### 3.1. Demographic Characteristics and Prevalence 

**Figure 1:**
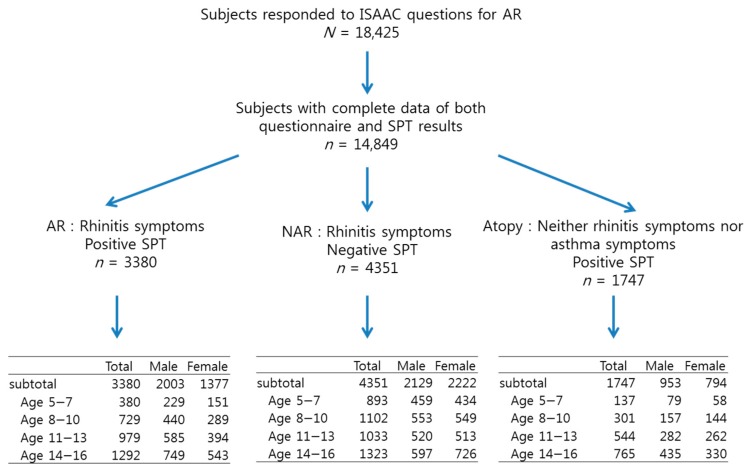
Flowchart of the study participants. Abbreviations: AR, allergic rhinitis; NAR, non-allergic rhinitis; SPT, skin prick test.

The flowchart of the study participants is illustrated in Figure 1. Among the total population of 18,425, we reviewed 14,849 subjects with complete data of both questionnaire and SPT results, aged 5−16 years old, of which 3380 were assigned to the AR group due to rhinitis symptoms with positive SPT, 4351 were assigned to the NAR group due to rhinitis symptoms with negative SPT, and 1747 were assigned to the atopy group due to no rhinitis or asthma symptoms with positive SPT. Of the population of 14,849, four age groups were constituted as follows: 2243 (15.1%) were 5−7 years, 3387 (22.8%) were 8−10 years, 4027 (27.1%) were 11−13 years, and5192 (35.0%) were 14−16 years. Of the total participants, 47.6% had had rhinitis symptoms based exclusively on the questionnaire. The total prevalence of AR was 21% (17.2% girls, 24.6% boys; *p* < 0.001 with significant difference between two compared groups) with a significant increase with age. The total prevalence of NAR was 26.5% (27.2% girls, 25.8% boys; *p* = 0.045) (Table 1). Boys had more symptoms of rhinitis and AR than girls in all age groups (data not shown, *p* < 0.001).

### 3.2. The Accuracy of the Questions 

The diagnostic accuracy of question on rhinitis “symptom, ever” was as follows: sensitivity of 57.5%, specificity of 58.4%, PPV of 44.2%, NPV of 70.6%, and accuracy of 58.1%. The questionnaire validity was compared among the different age groups, the highest sensitivity (63.2%) and NPV (82.0%), and the lowest PPV (30.6%) and accuracy (56.1%) were reported in the 5−7 years age group. The specificity, PPV, and accuracy tended to increase with age. The accuracy of “treatment, current” was the highest (63.8%), followed by “diagnosis, ever” (63.4%), “symptom, current” (59.2%), and “symptom, ever” (58.1%) (Table 2).

### 3.3. Comorbidity

**Figure 2:**
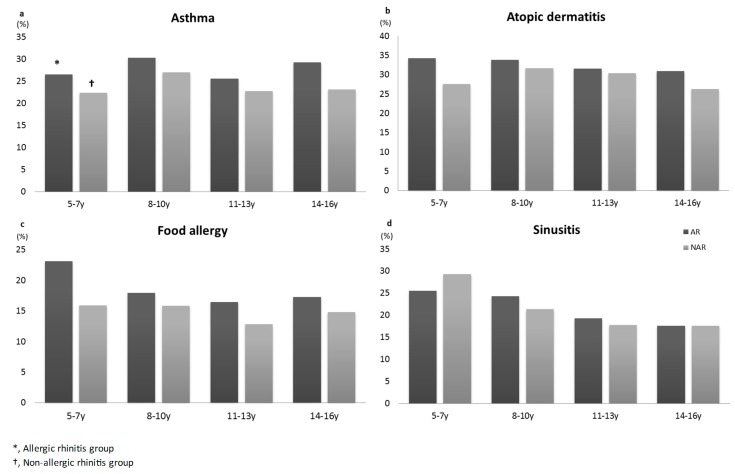
Comorbidities between children in different groups of AR and NAR. Abbreviations: AR, allergic rhinitis; NAR, non-allergic rhinitis.

**Figure 3:**
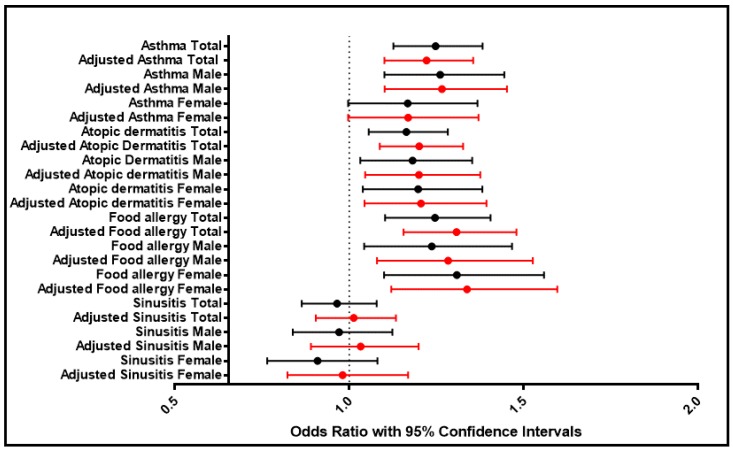
Confidence interval for odds ratio by allergic diseases. Odds ratios and adjusted odds ratios are displayed in black and red, respectively, *p* < 0.05.

Table 3 shows the results of logistic regression analyses. The aORs for age and sex were calculated to determine the association between other allergic diseases in the AR and NAR groups. The OR and aOR of having asthma, atopic dermatitis and food allergy were significantly greater in all of the AR subgroups relative to the NAR group (Table 3). However, sinusitis was equally prevalent in both groups (Figure 2 and Figure 3).

## 4. Discussion

The ISAAC core questions for rhinitis were incorporated into our questionnaire as they represent a widely accepted standardized tool for the assessment of the prevalence of rhinitis in children and adolescents [2]. However, as the validity of each question on rhinitis may differ per country, region, and age group, it is important to assess the validity of each question of the questionnaire in various subsets of the target population [7]. Although there are several good epidemiologic studies using the ISAAC questionnaires alone in Korean children [3,4], a few epidemiologic studies used the combination of questions on rhinitis symptoms and allergy testing for the identification of AR [5]. This study could adequately identify most of the AR patients, since the respiratory allergens included in this study were among those sensitized in the majority of the age groups in Korea [8].

There are a lack of epidemiological studies on the prevalence of NAR, probably because of an inconsistent definition and a lack of allergy skin testing. To our knowledge, this is the first population−based study on the prevalence of AR and NAR in children from various geographical locations in Korea. We found that the prevalence of AR and NAR was 21.0% and 26.5%, respectively, quite different from those in rhinitis patients who visited hospitals (AR 68.3% vs. NAR 14.5%) [9]. The prevalence of AR differed by sex (*p* < 0.001) and age (*p* < 0.001). The AR prevalence increased with age from 15.4% among those aged 5−7 years to 23% in the 14−16 years age group. Conversely, the prevalence of NAR in children was highest in those aged 5− years (34.9%), and decreased with age. We found that NAR was twice as common as AR in those aged 5−7 years (NAR 34.9% vs. AR 15.4%), which was different from those aged 14−16 years (NAR 23.4% vs. AR 23.0%).

In the present study, we were able to evaluate the validity of the ISAAC questionnaire for rhinitis with greater accuracy since it was performed in the general population, comprising many individuals from different regions. The question on the rhinitis “symptom, ever” in the ISAAC questionnaire was found to have moderate sensitivity and specificity (57.5% and 58.4%, respectively) with a high NPV (70.6%), low PPV (44.2%), and moderate accuracy (58.1%). The specificity and PPV were observed to increase with age, indicating that only 30% of 5 to 7−year−old children with “symptom, ever” and 50% of 11 to 16−year−old children are AR. Our results are different from those of a Swiss study on the validation of a rhinitis questionnaire (sensitivity 42.7%, specificity 77.5%, PPV 48.1%), which showed lower sensitivity and higher specificity compared to those in this study with Korean children [10]. Thus, the validity of the rhinitis question can be influenced by various factors including age, sex, region, and country. Despite the accuracy of “treatment, ever” being the highest among four questions, the PPV and the accuracy were 50.4% and 63.8%, respectively, suggesting allergy testing is required to define AR and NAR. 

Our findings demonstrate that the ISAAC questionnaire is moderately useful in detecting AR in the general population of children and adolescents, and highlights that further diagnostic tools including SPT are needed to confirm diagnoses, especially in younger children. This study also showed that the validity of a questionnaire might vary with age, indicating that the expected validity cannot be simply generalized for the whole pediatric age group.

We examined the prevalence of comorbid asthma, atopic dermatitis, food allergy, and sinusitis in the AR and NAR groups. We found that AR was more likely to be associated with asthma, atopic dermatitis, and food allergy, however, sinusitis was equally associated with both groups. In a Swedish study, comorbid allergic diseases associated in the AR versus the NAR groups (% in 4−year−old group, % in 8−year−old group) were asthma (28.0%, 25.5% vs. 12.7%, 11.0%), eczema (50.0%, 32.4% vs. 29.8%, 28.2%), and food hypersensitivity (38.1%, 51.1% vs. 17.7%, 13.4%) [11]. In a study analyzing the comorbidities for AR in 1275 Spanish children, the most frequent comorbidities were conjunctivitis (53.6%), asthma (49.5%), atopic dermatitis (40%), rhinosinusitis (26.1%), otitis media (23.8%), and adenoid hypertrophy (17.3%) [11].

This study demonstrates the strong association between the AR and atopic dermatitis in various pediatric age groups. The concept of the “atopic march” was developed to describe the progression of allergic disorders from atopic dermatitis to allergic rhinitis and asthma in children. This progression depends on various factors, such as the presence of filaggrin mutations and the time of onset, severity, and control of atopic dermatitis, which were found to be related to a higher risk for the development of AR [12]. Cases of AR triggered by food are uncommon, however, an Asian study detecting the relationship between food allergy and AR in children reported that the prevalence of food allergy in pediatric patients with AR is fairly high, which is consistent with our findings [13]. In the French arm of the European Community Respiratory Health Survey, current asthma was reported in 22.5% of the subjects with rhinitis and 4% of those without rhinitis [14]. However, increased prevalence of asthma has been reported both in children with AR and those with NAR, suggesting different endotypes of asthma symptoms [15]. Asthma has heterogeneous phenotypes, including atopic and nonatopic ones, thus, fractional exhaled nitric oxide (FeNO), separate from allergy testing, can be used as a diagnostic tool to distinguish AR and NAR [16]. Although our data showed that asthma was more prevalent in the AR group (28.1%), the NAR group also had a higher prevalence of asthma (23.9%) compared to general population (18.3%).

This study has some limitations. Due to the nature of epidemiological studies, anatomical deformities such as adenoid vegetation, or infectious causes such as chronic rhinosinusitis could not be excluded from the NAR group. Thus, the true prevalence of NAR may be overestimated. In addition, there should be mixed allergic and nonallergic rhinitis, which we were unable to identify in this study.

In summary, the prevalence of questionnaire−based AR was 47.6%. Utilizing both questions and SPT results, the prevalence of AR and NAR were 21% and 26.5%, respectively. Considering that the AR accuracy is about 60% based on the ISAAC questionnaire, further diagnostic tools including SPT should be considered to confirm diagnosis, especially in younger children.

## 5. Conclusions

This nationwide study showed prevalence rate of both AR and NAR among a general population of children aged 5−16 years by combining a modified questionnaire with SPT. Considering that the diagnostic accuracy based on the questionnaire alone was approximately 60%, the questionnaire survey therefore, overestimated the prevalence of AR in Korean children and adolescents. We hope that these findings extend our perspectives on the epidemiologic study in Korea.

## Figures and Tables

**Table 1:** Demographic characteristics and prevalence.

Rhinitis Questions	Rhinitis by Questions			AR by Questionnaire and Skin Prick Test *			NAR ^†^		
	Total (%) (*n* = 18,425)	Male (%) (*n* = 9292)	Female (%) (*n* = 9133)	Total (%) (*n* = 3380)	Male (%) (*n* = 2003)	Female (%) (*n* = 1377)	Total (%) (*n* = 4351)	Male (%) (*n* = 2129)	Female (%) (*n* = 2222)
**Symptom, ever ^‡^**									
Subtotal	47.6	50.6	44.4	21.0	24.6	17.2	26.5	25.8	27.2
Age 5−7	50.1	52.0	48.0	15.4	18.2	12.4	34.9	34.7	35.2
Age 8−10	49.5	54.4	44.6	19.6	23.8	15.4	29.6	29.9	29.3
Age 11−13	45.4	50.1	40.7	22.6	26.5	18.6	23.0	23.5	22.6
Age 14−16	46.6	47.7	45.6	23.0	26.6	19.4	23.4	20.9	26.0
**Symptom, current ^∫^**									
Subtotal	43.6	46.7	40.4	19.5	23.0	16.0	23.9	23.4	24.5
Age 5−7	47.1	49.0	45.0	14.4	16.9	11.7	32.7	32.2	33.2
Age 8−10	45.4	50.3	40.5	18.3	22.5	14.1	26.9	27.3	25.6
Age 11−13	41.5	46.1	36.7	21.3	25.1	17.3	20.7	21.2	20.2
Age 14−16	42.1	43.3	40.9	21.2	24.3	18.0	20.7	18.6	22.7
**Diagnosis, ever ^||^**									
Subtotal	29.4	32.4	26.2	14.5	17.5	11.4	14.7	14.9	14.5
Age 5−7	30.3	31.3	29.1	10.1	11.9	8.1	20.5	20.5	20.5
Age 8−10	32.3	37.3	27.2	14.5	18.1	11.0	17.8	19.5	16.1
Age 11−13	28.7	33.0	24.4	15.7	19.5	11.8	13.0	13.2	12.7
Age 14−16	27.2	29.0	25.5	15.5	18.1	12.8	11.5	10.7	12.4
**Treatment, current ^¶^**									
Subtotal	22.2	25.0	19.3	11.0	13.5	8.6	10.8	11.1	10.5
Age 5−7	25.7	27.2	24.0	8.7	10.4	6.8	17.5	17.6	17.3
Age 8−10	25.1	29.3	21.0	12.0	14.6	9.4	12.8	14.0	11.6
Age 11−13	21.7	26.1	17.3	12.0	15.4	8.5	9.6	10.3	8.9
Age 14−16	18.3	19.7	17.0	10.7	12.6	8.8	7.6	7.0	8.1

*, Positive response to rhinitis questions from Korean ISAAC questionnaires and ≥1 positive Result(s) of allergic skin prick test; ^†^, Positive response to rhinitis questions from Korean ISAAC questionnaires and negative results of allergic skin prick test; ^‡^, Children who have allergic rhinitis symptoms since birth; ^∫^, Children who have allergic rhinitis symptoms within 12 months; ^||^, Children who were diagnosed as having allergic rhinitis since birth; ^¶^, Children who underwent or were being treated for allergic rhinitis within 12 months; Abbreviations: AR, allergic rhinitis; NAR, non-allergic rhinitis; Abbreviations: AR, allergic rhinitis; NAR, non-allergic rhinitis.

**Table 2:** The validity of the questionnaire on rhinitis symptoms by skin prick test results.

	Sensitivity	Specificity	Positive Predictive Value	Negative Predictive Value	Youden’s Index (%) *	Accuracy (%) ^||^
**Symptom, ever ^†^**						
Subtotal	57.5	58.4	44.2	70.6	15.9	58.1
Age 5−7	63.2	53.8	30.6	82.0	17.0	56.1
Age 8−10	60.4	56.2	39.8	74.8	16.6	57.6
Age 11−13	56.4	61.6	49.5	67.9	18.0	59.5
Age 14−16	55.4	60.0	49.5	65.5	15.4	58.1
Symptom, current **						
Subtotal	53.5	62.4	44.9	70.1	15.9	59.2
Age 5−7	59.2	56.8	30.6	81.2	16.0	57.4
Age 8−10	56.4	60.2	40.4	74.3	16.6	59.0
Age 11−13	53.1	65.5	50.7	67.6	18.6	60.5
Age 14−16	51.0	64.7	50.6	65.1	15.7	59.0
**Diagnosis, ever ^‡^**						
Subtotal	39.8	76.9	49.6	69.1	16.7	63.4
Age 5−7	41.4	72.9	32.9	79.5	14.3	65.2
Age 8−10	44.9	73.7	44.9	73.6	18.5	64.3
Age 11−13	39.2	78.3	54.7	65.8	17.5	62.7
Age 14−16	37.3	80.3	57.3	64.3	17.6	62.4
**Treatment, current ^∫^**						
Subtotal	30.3	83.0	50.4	67.6	13.3	63.8
Age 5−7	35.7	76.9	33.2	78.8	12.6	66.9
Age 8−10	37.1	81.1	48.4	72.9	18.2	66.8
Age 11−13	29.9	83.9	55.5	64.2	13.9	62.3
Age 14−16	25.8	87.1	58.6	62.3	12.8	61.6

*, Index for rating diagnostic tests; ^†^, Children who have allergic rhinitis symptoms since birth; **, Children who have allergic rhinitis symptoms within 12 months; ^‡^, Children who were diagnosed as having allergic rhinitis since birth; ^∫^, Children who underwent or were being treated for allergic rhinitis within 12 months; ^||^, Probability of accurate diagnosis in total.

**Table 3:** Odds ratio for having comorbid disease in the AR group compared with NAR group.

		OR *	aOR ^†^	*p*-Value **	*p*-Value ^‡^	95% Confidence Interval ^∫^		95% Confidence Interval ^||^	
Asthma	Total	1.248	1.222	0.000	0.000	1.127	1.383	1.101	1.356
	Male	1.261	1.266	0.001	0.000	1.101	1.445	1.102	1.453
	Female	1.168	1.169	0.054	0.055	0.997	1.368	0.997	1.371
Atopic dermatitis	Total	1.164	1.201	0.002	0.000	1.056	1.283	1.088	1.327
	Male	1.182	1.200	0.016	0.009	1.032	1.353	1.046	1.376
	Female	1.198	1.206	0.013	0.010	1.039	1.382	1.044	1.394
Food allergy	Total	1.246	1.308	0.000	0.000	1.103	1.406	1.156	1.480
	Male	1.237	1.284	0.015	0.005	1.043	1.467	1.080	1.527
	Female	1.309	1.338	0.003	0.001	1.100	1.559	1.121	1.597
Sinusitis	Total	0.965	1.013	0.533	0.829	0.864	1.079	0.904	1.134
	Male	0.971	1.033	0.691	0.672	0.838	1.124	0.890	1.199
	Female	0.909	0.981	0.281	0.831	0.765	1.081	0.823	1.169

*, Unadjusted odds ratio; †, Adjusted odds ratio by age and gender; **, Logistic regression analysis without covariates; ^‡^, Logistic regression analysis with covariates (age and gender); ^∫^, Confidence interval for unadjusted odds ratio; ^||^, Confidence interval for adjusted odds ratio by age and gender; Abbreviations: aOR, adjusted odds ratio; OR, odds ratio.
